# Changes of repolarization parameters after left bundle branch area pacing and the association with echocardiographic response in heart failure patients

**DOI:** 10.3389/fphys.2022.912126

**Published:** 2022-08-04

**Authors:** Yao Li, Wenzhao Lu, Qingyun Hu, Chendi Cheng, Jinxuan Lin, Yu’an Zhou, Ruohan Chen, Yan Dai, Keping Chen, Shu Zhang

**Affiliations:** State Key Laboratory of Cardiovascular Disease, Arrhythmia Center, Fuwai Hospital, National Center for Cardiovascular Diseases, Chinese Academy of Medical Sciences and Peking Union Medical College, Beijing, China

**Keywords:** left bundle branch area pacing, repolarization, Tpeak—Tend, echocardiographic response, heart failure

## Abstract

**Background:** Left bundle branch area pacing (LBBAP) has become a safe and effective option for heart failure (HF) patients indicated for cardiac resynchronization therapy (CRT) and/or ventricular pacing, yet the response rate was only 70%. Repolarization parameters were demonstrated to be associated with cardiac mechanics and systolic function. This study aimed to investigate the effects of LBBAP on repolarization parameters and the potential association between those parameters and echocardiographic response.

**Methods and results:** A total of 59 HF patients undergoing successful LBBAP were consecutively included. QTc, Tpeak-Tend (TpTe), and TpTe/QTc were measured before and after the implantation. The results turned out that the dispersion of ventricular repolarization (DVR) improved after LBBAP among the total population. Although trends of repolarization parameters varied according to different QRS configurations at baseline, the post-implant parameters showed no significant difference between groups. The association between repolarization parameters and LBBAP response was then evaluated among patients with wide QRS. Multivariate analysis demonstrated that post-implant TpTe was the independent predictor of LBBAP response (p < 0.05). Receiver operating characteristic analysis indicated an area under the curve of 0.77 (95% CI, 0.60–0.93) with a cutoff value of 81.2 ms (p < 0.01). Patients with post-implant TpTe<81.2 ms had a significantly higher rate of echocardiographic response (93.3 vs. 44.4%, p < 0.01). Further subgroup analysis indicated that the predictive value of post-implant TpTe for LBBAP response was more significant in non-left bundle branch block (LBBB) patients than in LBBB patients.

**Conclusion:** LBBAP improved DVR significantly in HF patients. Post-implant TpTe was associated with the echocardiographic response after LBBAP among patients with wide QRS, especially for non-LBBB patients.

## Introduction

Cardiac resynchronization therapy (CRT) is an effective treatment for heart failure (HF) patients with cardiac dyssynchrony or indicated for ventricular pacing (Epstein et al., 2013). Recently, left bundle branch area pacing (LBBAP) has been reported as a safe and effective option for CRT delivery (Vijayaraman et al., 2021), which achieved better electrical synchrony and even more promising echocardiographic and clinical outcomes than traditional biventricular pacing (BiVP) (Li et al., 2020; Strocchi et al., 2020). However, a large proportion of patients still showed LBBAP non-response, up to around 30% (Vijayaraman et al., 2021). Data on predictors for LBBAP response or non-response are quite limited to date.

Ventricular repolarization was demonstrated to be significantly associated with myocardial mechanics and systolic function in the last decade (Sauer et al., 2012; Sauer et al., 2014; Prenner et al., 2016). Indices of dispersion of ventricular repolarization (DVR) were even reported to be correlated with BiVP response in HF patients. For example, Yu et al. found that baseline Tpeak-Tend (TpTe)/QTc could predict CRT non-response among BiVP patients (Yu et al., 2017). The relationship between repolarization parameters and LBBAP response has never been studied yet.

The current study aimed to evaluate the changes in repolarization parameters after LBBAP, and further determine the potential association between baseline and post-implant repolarization parameters and echocardiographic response.

## Methods

### Study population

HF patients undergoing successful LBBAP implantation at our center from December 2017 to February 2022 were consecutively and retrospectively enrolled in the study. The inclusion criteria were as follows: 1) New York Heart Association II-IV despite optimal guideline-based medication for at least 3 months; 2) left ventricular ejection fraction (LVEF) < 50%; 3) indications for CRT implantation and/or ventricular pacing. All patients had written informed consents for the operation and clinical data use. This study was approved by the hospital Institutional Review Board.

### Procedure details

Procedure details of LBBAP were elaborated on in previous studies (Chen et al., 2019; Li et al., 2019; Lin et al., 2021). Briefly, the Select Secure pacing lead (Model 3830 69 cm, Medtronic Inc., Minneapolis, MN, United States) was screwed perpendicularly into the ventricular septum towards the left bundle branch (LBB) area under fluoroscopic RAO30° through a fixed-curve sheath (C315 HIS, Medtronic Inc., Minneapolis, MN, United States). During the whole process, unipolar-tip paced QRS configuration and pacing impedance were monitored closely along with measurement of stimulus to peak left ventricular activation time (stim-LVAT). The criteria for LBB area capture were shown below (Lin et al., 2020; Vijayaraman et al., 2021). The unipolar-tip paced QRS showed right bundle branch block (RBBB) configuration or correction of left bundle branch block (LBBB) along with at least one of the following findings:1) transition from non-selective LBB capture to selective LBB capture as the output decreased at the same site; 2) transition from non-selective LBB capture to left ventricular (LV) septal capture as the output decreased at the same site (prolongation of stim-LVAT ≥ 10 ms); 3) short and constant stim-LVAT despite the change of output (stim-LVAT ≤ 90 ms).

### Measurement of electrocardiogram (ECG) parameters

ECGs under intrinsic rhythm and ventricular-paced rhythm around 24 h after the implantation were recorded, respectively, for each patient. Trained assessor masked to treatment allocation did all the ECG metrics. QRS duration (QRSd) was measured from the earliest onset throughout all leads to the latest offset. QT interval and TpTe interval were measured in V5 lead for three beats during sinus rhythm and five beats during atrial fibrillation (Af), then the average was taken (Antzelevitch et al., 2007; Tooley et al., 2019). QT interval was measured from the onset of the QRS complex to the end of the T-wave, defined as the point at which a tangent to the maximal downslope of the descending limb of the T-wave crossed the isoelectric baseline (Castro Hevia et al., 2006). QTc was the correction the of QT interval for HR and the Bazett formula was used (Lee et al., 2014; Cho et al., 2016; Vandenberk et al., 2016). TpTe was the difference between QT interval and QTpeak (distance from the onset of QRS complex to the peak of T wave). QTpeak was measured to the nadir the of T wave the if T wave was negative or biphasic (Emori and Antzelevitch, 2001; Antzelevitch et al., 2007; Itoh et al., 2013). Specifically, TpTe in the case of a negative–positive T wave was the interval between the nadir of the initial negative T wave and the end of the T wave.

### Echocardiography

Transthoracic 2-dimensional echocardiography was performed within 1 week before CRT implantation and at least 3 months after implantation. LVEF was quantified using the biplane Simpson method, and LVEF and left ventricular end-diastolic diameter (LVEDD) were recorded. A positive echocardiographic response was defined as a ≥5% increase in LVEF between baseline and follow-up echocardiography (Vijayaraman et al., 2021). Super-response was achieved when LVEF increased by at least 20% or improved to >50% in patients with baseline LVEF ≤35% (Ellenbogen and Huizar, 2012).

### Statistics

All statistical analyses were performed with SPSS software 25.0. The continuous variables, expressed as mean ± standard deviation (normal distribution) or median (interquartile range) (non-normal distribution), were analyzed with a student’s t-test or non-parametric test (Mann–Whitney test or Wilcoxon signed rank test) between two groups while with one-way ANOVA or Kruskal–Wallis test between three groups. Shapiro–Wilk was used for the normality test. Categorical variables were presented as numbers and percentages, and statistical significance was assessed by the Chi-square test or Fisher’s exact test. Stepwise multivariate logistic regression analysis was used to identify the independent predictors for LBBAP response (univariate factors presenting *p* < 0.15 were included). The optimal cut-off point for response predictor was obtained by receiver operating characteristic (ROC) curve analysis based on maximal Youden index (sensitivity—[1—specificity)). All tests were two-tailed, and a *p* value <0.05 was considered statistically significant.

## Results

### Baseline and procedure characteristics

A total of 59 HF patients with a mean LVEF of 33.7 ± 6.7% were included in the study. The median age of the patients was 64.0 years (67.8% men). Ischemia cardiomyopathy and Af were observed in 15.3% and 25.4% of the patients, respectively. 81.4% of the patients had QRS ≥130 ms [(54.2% had LBBB, 13.6% had intraventricular conduction defects, and 13.6% had prior right ventricular paced (RVP) rhythm], while the remaining 18.6% of patients had narrow QRS. (Table 1).

**TABLE 1:** Baseline characteristics of study population.

Variables	LBBAP group (*n* = 59)
Age (years)	64.0 (56.0, 70.0)
Gender (%, male)	40 (67.8)
Atrial fibrillation (%)	15 (25.4)
Hypertension (%)	30 (50.8)
Diabetes (%)	11 (18.6)
CAD (%)	25 (42.4)
ICM (%)	9 (15.3)
Hyperlipidemia (%)	29 (49.2)
CKD (%)	3 (5.0)
LVEF (%)	33.7 ± 6.7
LVEDD (mm)	64.5 ± 9.3
QRS morphology	-
LBBB	32 (54.2)
IVCD	8 (13.6)
RVP	8 (13.6)
Narrow QRS (<130 ms)	11 (18.6)
ACEI/ARB (%)	48 (81.4)
*ß* blockers (%)	58 (98.3)
Amiodarone (%)	10 (16.9)

ACEI, angiotensin-converting enzyme inhibitor; ARB, angiotensin receptor blocker; CAD, coronary artery disease; CKD, chronic kidney disease; ICM, ischemia cardiomyopathy; IVCD, intraventricular conduction defects; LBBAP, left bundle branch area pacing; LBBB, left bundle branch block; LVEDD, left ventricular end‐diastolic diameter; LVEF, left ventricular ejection fraction; RVP, right ventricular pacing.

Procedure-related characteristics were recorded in Table 2. All patients achieved favorable pacing parameters during the procedure. After a median follow-up period of 4 months (range 3.0–8.0 months), R wave amplitude remained stable while pacing threshold increased slightly from 0.5 (0.5, 0.5) to 0.5 (0.5, 0.8) v/0.4 ms (*p* < 0.01) and pacing impedance decreased from 516.9 ± 133.8 to 456.4 ± 81.8 Ω (*p* < 0.001). The pacing percentage was 99.8 (97.2, 100) % among the population.

**TABLE 2:** Procedure characteristics (*n* = 59).

Pacing parameters	Baseline	Follow-up	*p* value
R wave amplitude (mV)	9.2 ± 4.3	10.7 ± 4.9	0.07
Capture threshold (V@0.4ms)	0.5 (0.5, 0.5)	0.5 (0.5, 0.8)	0.002**
Pacing impedance(Ω)	516.9 ± 133.8	456.4 ± 81.8	<0.001***
Stim-LVAT (ms)	70 (63, 80)	NA	NA

NA, not applicable; Stim-LVAT, stimulus to peak left ventricular activation time.

*p < 0.05, **p < 0.01, ***p < 0.001.

### Changes in ECG parameters after the implantation

**FIGURE 1:**
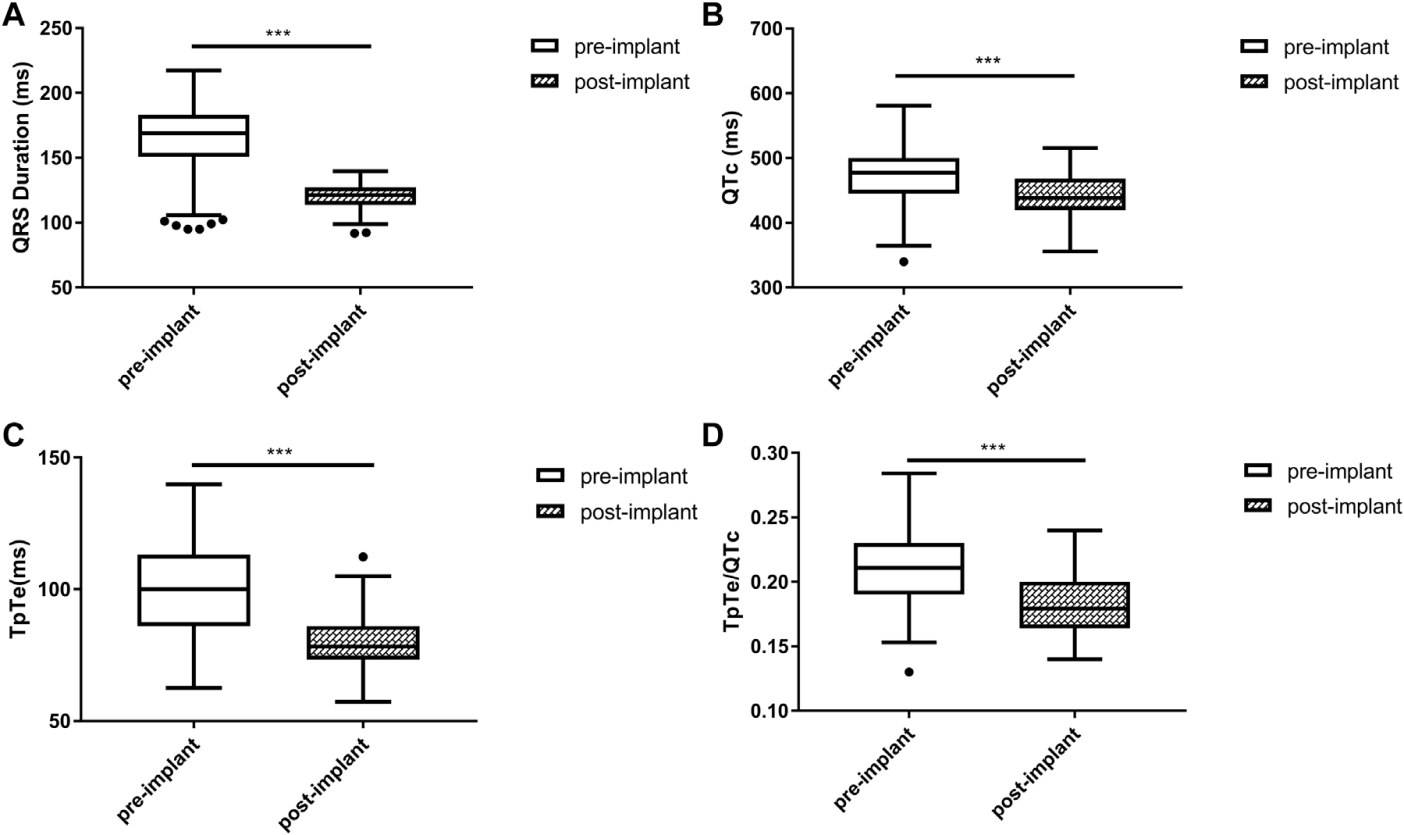
Changes in QRS duration **(A)**, QTc **(B)**, TpTe **(C)**, TpTe/QTc **(D)** before and after LBBAP in HF patients (*n* = 59). (Box plots exhibited the smallest nonoutlier, first quartile, median, third quartile, and largest non-outlier of each variable from bottom to top. HF, heart failure; LBBAP, left bundle branch area pacing; QTc, QT corrected measurement; TpTe, Tpeak-end interval. *p < 0.05, **p < 0.01, ***p < 0.001).

Biphasic T wave with the positive component following the negative component was observed in 12 (20.3%) patients. For the entire study population, QRSd significantly decreased after the implantation [121.3 (113.8, 127.2) vs. 168.9 (150.8, 183.0) ms, *p* < 0.001]. Post-implant repolarization parameters such as QTc (442.7 ± 35.8 vs. 469.8 ± 51.5 ms, *p* < 0.001), TpTe [78.3 (73.3, 86.0) vs. 100.0 (86.0, 113.1) ms, *p* < 0.001] and TpTe/QTc (0.18 ± 0.02 vs. 0.21 ± 0.03, *p* < 0.001) also decreased compared with baseline. (Figure 1).

**FIGURE 2:**
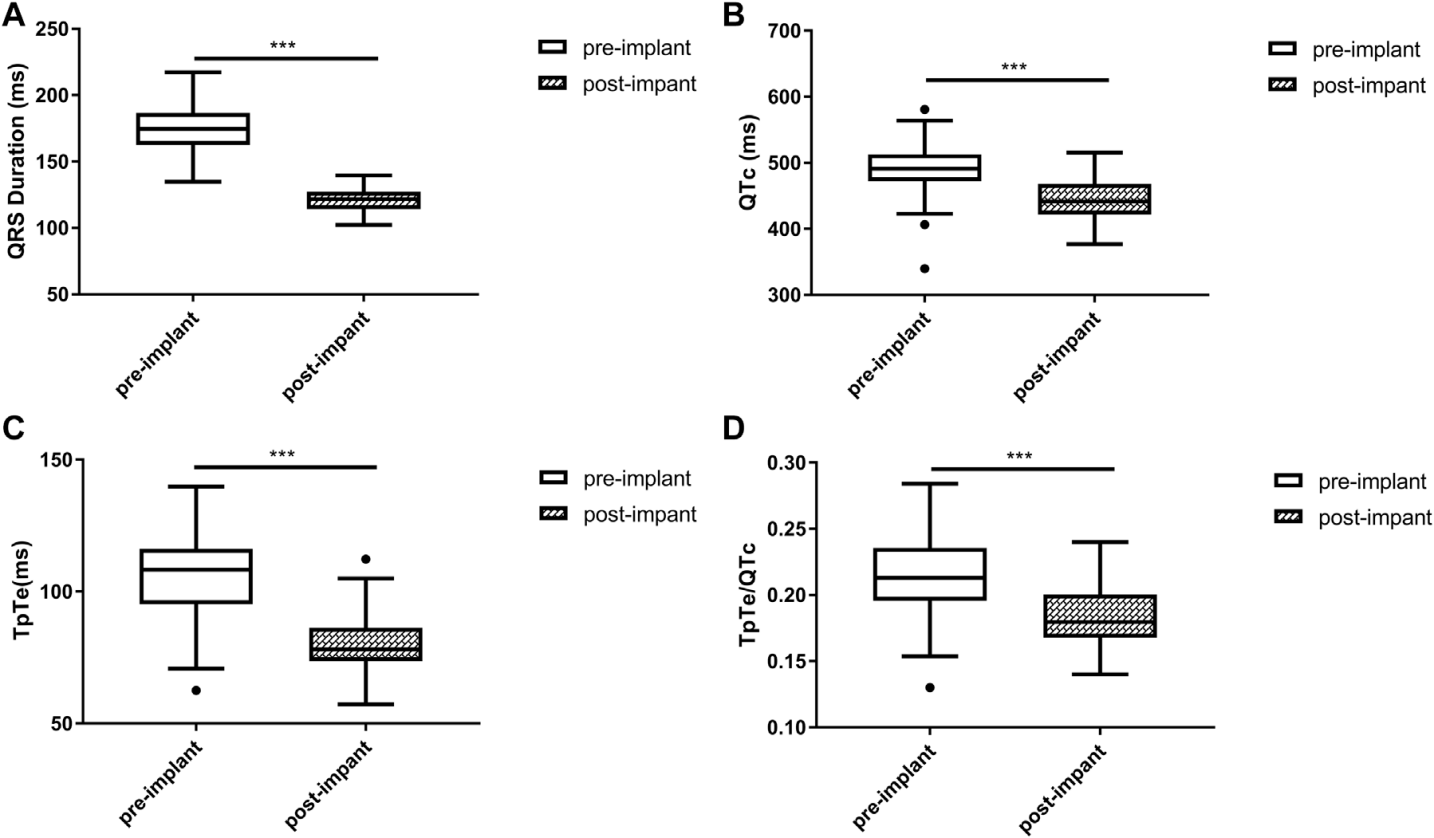
Changes in QRS duration **(A)**, QTc **(B)**, TpTe **(C)**, TpTe/QTc **(D)** before and after LBBAP in patients with wide QRS but not paced rhythm (*n* = 40). (Box plots exhibited the smallest nonoutlier, first quartile, median, third quartile, and largest non-outlier of each variable from bottom to top. LBBAP left bundle branch area pacing; QTc, QT corrected measurement; TpTe, Tpeak-Tend interval. *p < 0.05, **p < 0.01, ***p < 0.001).

**FIGURE 3:**
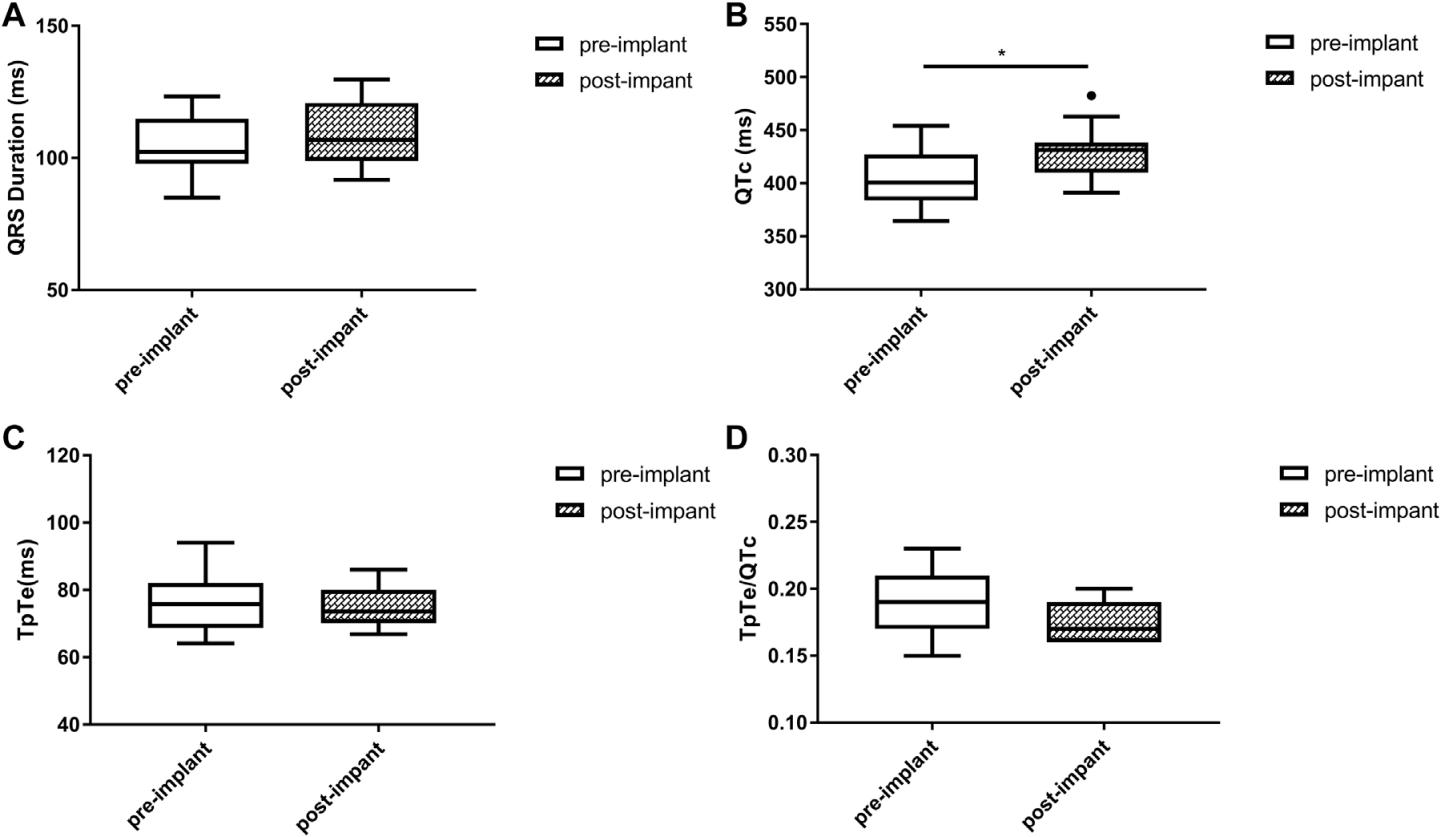
Changes in QRS duration **(A)**, QTc **(B)**, TpTe **(C)**, TpTe/QTc **(D)** before and after LBBAP in patients with baseline narrow QRS (*n* = 11). (Box plots exhibited the smallest nonoutlier, first quartile, median, third quartile, and largest non-outlier of each variable from bottom to top. LBBAP left bundle branch area pacing; QTc, QT corrected measurement; TpTe, Tpeak-Tend interval. *p < 0.05, **p < 0.01, ***p < 0.001).

**FIGURE 4:**
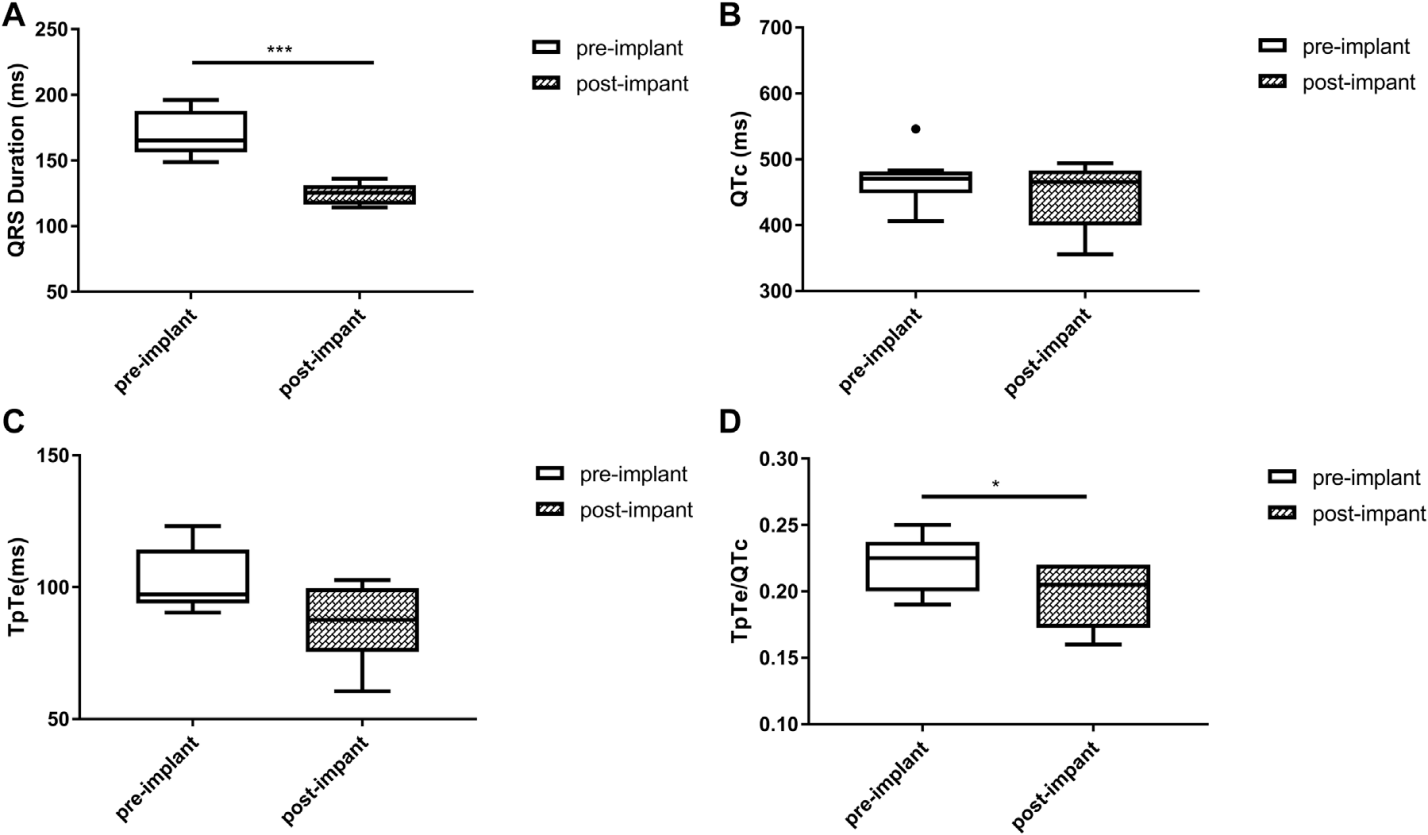
Changes in QRS duration **(A)**, QTc **(B)**, TpTe **(C)**, TpTe/QTc **(D)** before and after LBBAP in patients with prior right ventricular paced rhythm (*n* = 8). (Box plots exhibited the smallest nonoutlier, first quartile, median, third quartile, and largest non-outlier of each variable from bottom to top. LBBAP left bundle branch area pacing; QTc, QT corrected measurement; TpTe, Tpeak-Tend interval. *p < 0.05, **p < 0.01, ***p < 0.001).

Baseline QRSd and prior RVP history (CRT upgrade) are known to influence ventricular repolarization after traditional BiVP (Chalil et al., 2006; Lellouche et al., 2007). Therefore, we further analyzed the changes in ECG parameters after LBBAP according to baseline ECG patterns. For patients with QRSd ≥130 ms, QRSd (120.9 ± 8.7 vs. 174.5 ± 17.1 ms, *p* < 0.001) and the repolarization parameters [QTc, 445.5 ± 35.2 vs. 487.9 ± 43.5 ms, *p* < 0.001; TpTe, 78.1 (73.6, 86.3) vs. 108.3 (95.2, 116.2) ms, *p* < 0.001; TpTe/QTc, 0.18 ± 0.02 vs. 0.22 ± 0.03, *p* < 0.001] were all significantly decreased after LBBAP (Figure 2). For patients with QRSd <130 ms, QRSd, TpTe, and TpTe/QTc were similar before and after the implantation, only QTc (430.0 ± 25.9 vs. 404.4 ± 29.9 ms, *p* < 0.05) was significantly increased after the implantation (Figure 3). For upgrade patients, ECGs recorded during baseline RVP and post-implant LBBAP were compared. The results turned out that post-implant QRSd (124.7 ± 7.9 vs. 169.0 ± 17.4 ms, *p* < 0.001) and TpTe/QTc (0.19 ± 0.02 vs. 0.22 ± 0.02, *p* < 0.05) significantly decreased; while no significant changes in QTc and TpTe were observed (Figure 4). In addition, although baseline ECG data were statistically different between the three groups, repolarization parameters after the implantation showed no significant difference (Table 3).

**TABLE 3:** Comparison of electrocardiographic data between patients with different QRS configuration at baseline.

	QRS>130 ms (a, *n* = 40)	QRS≤130 ms (b, *n* = 11)	Paced QRS (c, *n* = 8)	*p* value		
				(a) vs. (b)	(a) vs. (c)	(b) vs. (c)
Baseline QRSd (ms)	174.5 ± 17.1	105.7 ± 9.8	169.0 ± 17.4	<0.001***	0.38	<0.001***
Baseline QTc (ms)	487.9 ± 43.5	404.4 ± 29.9	469.2 ± 39.5	<0.001***	0.24	0.001**
Baseline TpTe (ms)	108.3 (95.2, 116.2)	75.8 (68.7, 82.0)	97.3 (93.8, 114.3)	<0.001***	0.999	0.01*
Baseline TpTe/QTc	0.22 ± 0.03	0.19 ± 0.02	0.22 ± 0.02	0.01*	0.72	0.03*
Post-impant QRSd (ms)	120.9 ± 8.7	109.0 ± 12.7	124.7 ± 7.9	0.001**	0.29	0.001**
Post-impant QTc (ms)	445.5 ± 35.2	430.0 ± 25.9	446.2 ± 49.8	0.44		
Post-impant TpTe (ms)	78.2 (73.6, 86.3)	73.6 (70.1, 80.0)	87.6 (75.5, 99.6)	0.052		
Post-impant TpTe/QTc	0.18 (0.17,0.20)	0.17 (0.16, 0.19)	0.20 (0.17,0.21)	0.23		

QRSd, QRS duration; QTc, corrected QT interval; TpTe, Tpeak‐Tend interval.

*p < 0.05, **p < 0.01, ***p < 0.001.

### Echocardiographic improvement after LBBAP

After a median follow-up period of 4 months, overall LVEF improved significantly from 33.7 ± 6.7% to 46.6 ± 11.0%, while LVEDD decreased from 63.0 (57.0, 71.0) mm to 55.0 (53.0, 63.0) mm (*p* < 0.001). Echocardiographic response was noted in 44 (74.6%) patients for the whole population. Specifically, the response rate was 72.7% (8/11) for patients with QRS<130 ms while 75% (36/48) for patients with wide QRS. Baseline LVEF and LVEDD were similar between responders and non-responders, while follow-up LVEF [52.5 (43.5, 56.8) vs. 36.0 (28.0, 44.0) %, *p* < 0.001] was significantly higher and LVEDD [54.0 (50.3, 58.8) vs. 63.0 (55.0, 69.0) mm, *p* < 0.01] was significantly smaller in responders. The absolute improvement in LVEF was also significantly higher in responders than non-responders [16.0 (8.0, 23.0) vs. 2.0 (-2.0, 4.0) %, *p* < 0.001]. Super-response was noted in 18 (30.5%) patients, and the improvement in LVEF in this subgroup was even higher (26.4 ± 5.8 vs. 7.0 ± 6.0%,*p* < 0.001).

### The association between ECG parameters and CRT response

The association between ECG parameters and CRT response in patients with wide QRS (QRSd ≥130 ms with paced rhythm or not) was evaluated. A comparison of baseline characteristics between responders and non-responders was illustrated in Table 4. Stepwise multivariable analysis was performed including factors presenting *p* < 0.15 in univariate analysis (amiodarone, post-implant QRSd, post-implant TpTe, and post-implant QTc) and previously identified correlation factors (LBBB and LVEDD), and the result turned out that post-implant TpTe (odds ratios: 0.887, 95% confidence interval: 0.802 to 0.982, *p* < 0.05), amiodarone, and baseline LVEDD were independently associated with LBBAP response (Table 5). To be noted, post-implant TpTe/QTc with *p* = 0.08 was excluded from the multivariate model due to the collinearity problem.

**TABLE 4:** Comparison of clinical, electro-cardiographic and echocardiographic data between responders and non-responders with QRS >130 ms.

Variables	Responder (*n* = 36)	Non-responder (*n* = 12)	*p* value
Baseline clinical characteristics	-	-	-
Age (years)	61.4 ± 13.6	60.6 ± 9.8	0.85
Gender (%, male)	22 (61.1)	8 (66.7)	0.999
Atrial fibrillation (%)	4 (11.1)	4 (33.3)	0.18
Hypertension (%)	18 (50)	7 (58.3)	0.62
Diabetes (%)	6 (16.7)	2 (16.7)	0.99
CAD (%)	16 (44.4)	5 (41.7)	0.87
ICM (%)	5 (13.9)	1 (8.3)	0.99
Hyperlipidemia (%)	18 (50.0)	7 (58.3)	0.62
CKD (%)	3 (8.3)	0 (0.0)	0.56
ACEI/ARB (%)	29 (80.6)	9 (75.0)	0.999
*ß* blockers (%)	36 (100.0)	12 (100.0)	0.999
Amiodarone (%)	5 (13.9)	5 (41.7)	0.10
Electrocardiographic parameters	-	-	-
QRS morphology	-	-	-
LBBB (%)	26 (72.2)	6 (50.0)	0.29
Baseline QRSd (ms)	173.2 ± 14.8	173.6 ± 23.6	0.99
Baseline QTc (ms)	485.3 ± 43.0	483.2 ± 45.0	0.88
Baseline TpTe (ms)	101.1 (94.3, 114.4)	108.6 (99.9, 118.2)	0.40
Baseline TpTe/QTc	0.21 (0.20, 0.22)	0.23 (0.21, 0.24)	0.16
Post-impant QRSd (ms)	120.3 ± 9.0	125.0 ± 6.9	0.10
Post-impant QTc (ms)	440.4 ± 37.0	461.3 ± 37.1	0.09
Post-impant TpTe (ms)	77.2 (73.3, 81.0)	90.1 (82.0, 99.3)	0.006**
Post-impant TpTe/QTc	0.18 ± 0.02	0.19 ± 0.02	0.08
Echocardiographic parameters	-	-	-
Baseline LVEF (%)	32.8 ± 6.5	35.1 ± 6.5	0.24
Baseline LVEDD (mm)	65.0 (59.0, 71.8)	64.0 (60.3, 70.5)	0.68
Follow-up LVEF (%)	49.0 ± 9.4	36.3 ± 8.6	<0.001***
Follow-up LVEDD (mm)	54.5 (50.3, 59.8)	64.0 (56.3, 70.5)	0.003**
Delta LVEF (%)	14.5 (8.0, 23.0)	2.0 (−2.0, 4.0)	<0.001***

ACEI, angiotensin-converting enzyme inhibitor; ARB, angiotensin receptor blocker; CAD, coronary artery disease; CKD, chronic kidney disease; ICM, ischemia cardiomyopathy; LBBB, left bundle branch block; LVEDD, left ventricular end‐diastolic diameter; LVEF, left ventricular ejection fraction; QRSd, QRS duration; QTc, corrected QT interval; TpTe, Tpeak‐Tend interval.

*p < 0.05, **p < 0.01, ***p < 0.001.

**TABLE 5:** Multivariable logistic regression analysis of factors associated with echocardiographic response among patients with QRS >130 ms (*n* = 48).

Factor	B	SE	P	OR	95% CI
LBBB	1.29	1.14	0.26	3.643	0.391–33.949
LVEDD	−0.14	0.06	0.03*	0.866	0.764–0.982
Amiodarone	−3.30	1.36	0.02*	0.037	0.003–0.528
Post-implant QRSd	−0.08	0.06	0.20	0.923	0.818–1.042
Post-implant TpTe	−0.12	0.05	0.02*	0.887	0.802–0.982
Post-implant QTc	0.005	0.02	0.74	1.005	0.976–1.036
Constant	28.25	12.26	0.02*	NA	NA

LBBB, left bundle branch block; LVEDD, left ventricular end‐diastolic diameter; LVEF, left ventricular ejection fraction; NA, not applicable; QRSd, QRS duration; QTc, corrected QT interval; TpTe, Tpeak‐Tend interval.

*p < 0.05.

**FIGURE 5:**
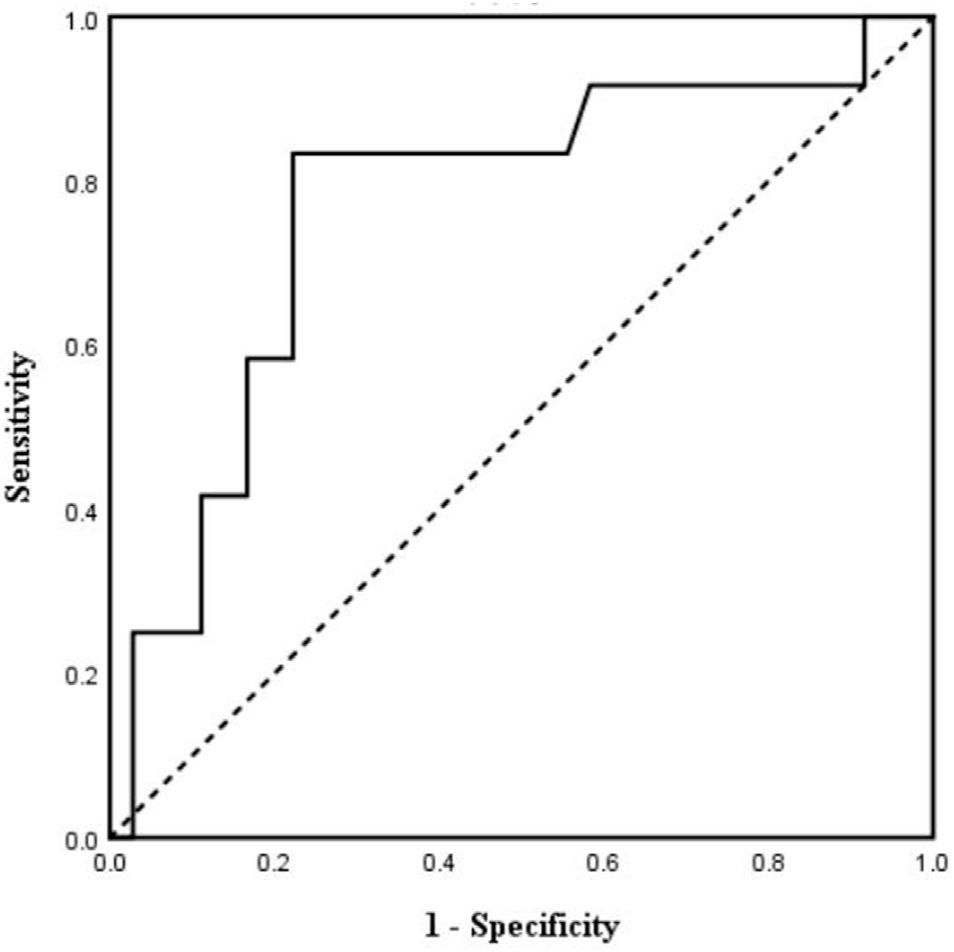
Receiver operating characteristic curve analysis of post-implant TpTe according to echocardiographic response (*n* = 48). TpTe: Tpeak-Tend interval.

ROC curve analysis was then performed. The result showed an area under the curve of 0.77 with 95% confidential interval of 0.60–0.93 (*p* < 0.01) (Figure 5). Sensitivity and specificity were 83.3% and 77.8% respectively with a cutoff value of 81.2 ms for post-implant TpTe predicting LBBAP echocardiographic response. The positive and negative predictive values of post-implant TpTe were 93.3% and 55.6%, respectively. Patients with post-implant TpTe shorter than 81.2 ms had more significant increase in LVEF [12.5 (7.0, 23.0) vs. 4.0 (1.0, 14.0) %, *p* < 0.05] than others. The rate of echocardiographic response was also significantly higher in this subgroup of patients (93.3 vs. 44.4%, *p* < 0.01).

Subgroup analysis was then performed among patients with or without LBBB at baseline. Among the 16 patients with non-LBBB, post-implant TpTe <81.2 ms showed a significantly higher rate of LBBAP response (100% vs. 14.3%, *p* < 0.01). However, of the remaining 32 patients with LBBB at baseline, the response rate seemed higher in patients with shorter TpTe but without statistical significance (90.5 vs. 63.6%, *p* = 0.07). Of note, no significant difference was observed in post-implant TpTe between responders and non-responders among patients with narrow QRS at baseline (74.6 ± 9.9 vs. 75.2 ± 5.6 ms, *p* > 0.05).

## Discussion

The study found that DVR improved after LBBAP among the total HF population. Although trends of repolarization parameters varied according to different QRS configurations at baseline, the post-implant parameters were similar between groups. In addition, LBBAP caused significant echocardiographic improvement after a median follow-up period of 4 months, and post-implant TpTe was significantly associated with the echocardiographic response after LBBAP among patients with wide QRS.

### Effects of LBBAP on repolarization parameters in HF patients

QTc, TpTe, and TpTe/QTc are all simple and accurate ECG markers of DVR (Shimizu and Antzelevitch, 1998; Prenner et al., 2016). These parameters are not only predictors of ventricular arrhythmias (VAs) and sudden cardiac death after CRT implantation but also related to myocardial mechanics (myocardial contraction and dilation) (Chalil et al., 2006; Sauer et al., 2014; Cvijic et al., 2018). Recently, Ponnusamy and Vijayaraman (2021) found that QTc, TpTe, and TpTe/QTc decreased immediately after LBBAP in 13 patients with LBBB-induced cardiomyopathy, preliminarily indicating the improvement of DVR after LBBAP. Our study analyzed the changes in repolarization parameters after LBBAP in the largest HF population to date, and the results turned out that QTc, TpTe, and TpTe/QTc significantly decreased, consistent with Ponnusamy’s study. Those results possibly suggested lower risks of VAs and better myocardial mechanics after LBBAP in HF patients and further studies were in need to prove this.

To be noted, baseline QRS configurations of the population were heterogeneous in our study. QRS configuration was known to influence repolarization parameters (Chalil et al., 2006; Lellouche et al., 2007), thus the study further analyzed those parameters in terms of baseline QRS patterns. In patients with QRS ≥130 ms, all three repolarization parameters decreased significantly after LBBAP, indicating the definite improvement of DVR in this subgroup of patients. In the other two groups, the changes of QTc were slightly discordant with the changes of TpTe and TpTe/QTc. Since TpTe reflected more accurately DVR than QTc (Yan and Antzelevitch, 1998), it was thus concluded that DVR improved in patients with prior RV-paced rhythm while barely deteriorated in patients with narrow QRS after LBBAP. Those results suggested the protective effects of LBBAP on repolarization stability in HF patients despite baseline QRS morphology. A similar study on BiVP found that DVR increased significantly in patients with LBBB or narrow QRS (Lellouche et al., 2007). Therefore, LBBAP might show prominent edges over BiVP concerning repolarization stability in HF patients indicated for CRT.

The mechanisms underlying the changes in DVR after LBBAP remain unclear. Mechano-electrical feedback, an established mechanism whereby myocardial strain causes changes in electrophysiological parameters, may partly account for this (Orini et al., 2017). Specifically, the reduction of ventricular wall stress and the improvement of ventricular synchrony could reverse the remodeling of repolarization-related potassium and calcium channels (Aiba and Tomaselli, 2012), which may be the molecular basis for the improvement of DVR after LBBAP in patients with wide QRS. Since LBBAP could achieve quite narrow QRSd and near-physiological ventricular synchrony (Strocchi et al., 2020), DVR barely deteriorated in patients with narrow QRSd at baseline.

### Post-implant TpTe was associated with the echocardiographic response after LBBAP

LV reverse remodeling of HF patients after LBBAP has been demonstrated by several studies (Huang W. et al., 2020; Li et al., 2020; Vijayaraman et al., 2021). The population in this study achieved an echocardiographic response rate of 74.6% after a median follow-up period of 4 months. However, two recent studies reported a much higher response rate of 88.9% (Huang W. et al., 2020)and 92% (Li et al., 2020), respectively. Baseline QRS configuration and definition of echocardiographic response may account for this discrepancy. For example, both studies mentioned above merely included patients with LBBB at baseline, which has been demonstrated as an independent predictor of LBBAP response (Vijayaraman et al., 2021). Yet baseline QRS pattern was heterogeneous in our study, only 54.2% were LBBB. Consistent with our results, Vijayaraman et al. reported a response rate of 73% in a population with various baseline QRS configurations (Vijayaraman et al., 2021).

Data on predictors for LBBAP response or non-response are quite limited to date. Recently, LBBB and smaller LVEDD were reported as independent predictors of echocardiographic response after LBBAP (Vijayaraman et al., 2021). Yet even in an LBBB population, around 10% of cases showed LBBAP was non-responsive (Huang W. et al., 2020; Li et al., 2020). More favorable predictors remain a matter of investigation. Ventricular repolarization parameters were related to cardiac mechanics and systolic function. Sauer et al. reported an independent relationship between TpTe and radial SD-tPkS (measuring contraction duration heterogeneity on echocardiography) (Sauer et al., 2014). In addition, DVR was found to be significantly correlated with impaired LVEF (Huang H. C. et al., 2020). However, whether repolarization parameters were associated with LBBAP response remains unclear. In the current cohort, post-implant TpTe was proved to be the independent predictor of echocardiographic response in addition to previously identified LVEDD among patients with wide QRS. Further ROC analysis determined a cut-off value of 81.2 ms in predicting echocardiographic response (sensitivity 83.3%, specificity77.8%, *p* < 0.01). Post-implant TpTe <81.2 ms had a high positive predictive value (93.3%) in LBBAP response, although an ECG not meeting the aforementioned criteria did not guarantee LBBAP non-response (negative predictive value 55.6%). These results indicated the significant association between post-implant TpTe and echocardiographic response after LBBAP in HF patients. Of note, amiodarone was also significant in the multivariate model with an OR of 0.037, consistent with the previous finding that amiodarone was related to adverse outcomes in patients upgraded to CRT-defibrillators (Adelstein et al., 2019). These two new predictors may further optimize LBBAP response via post-implant management.

A previous study has reported baseline LBBB as an independent predictor of LBBAP response (Vijayaraman et al., 2021). Consistently, there were more LBBB patients in responders than non-responders in our study, although without statistical significance (responders vs. non-responders: 72.2% vs. 50%). Since non-LBBB tends to result in a higher rate of non-response and worse prognosis, it is of vital clinical significance to improve response in this subgroup of patients. In the current study, post-implant TpTe <81.2 ms showed a significantly higher rate of LBBAP response among patients with non-LBBB (100% vs. 14.3%, *p* < 0.01). The result implied that a high percentage of non-LBBB patients may respond to LBBAP if post-implant TpTe criteria were met through post-operative ECG management. For LBBB patients, the TpTe criteria seemed to exert similar influence although without statistical significance (90.5 vs. 63.6%, *p* = 0.07). Thus, the effects of post-implant TpTe in predicting echocardiographic response may be more prominent in non-LBBB patients than LBBB patients although larger-sample trials were warranted to verify this. In addition, no association between post-implant TpTe and LBBAP response among patients with narrow QRSd was observed, thus the predictive value of this ECG marker could not be applied to these patients.

This was the first study to demonstrate the potential value of repolarization parameters in predicting LBBAP echocardiographic response, although the underlying mechanisms remained to be further studied. Electromechanical coupling and calcium handling may play a role (Prenner et al., 2016). If our data are to be confirmed by larger prospective studies, post-implant TpTe should be considered for future management of CRT recipients in addition to paced QRSd and QRS morphology to further improve response.

However, the study still had several limitations. First, this was a retrospective, single-center study with small sample size, thus our results needed to be confirmed by a larger, prospective study. Second, echocardiogram measurements were not performed by two independent investigators due to the retrospective nature of the study. Finally, only echocardiographic response was assessed in the study, and it would be more desirable to assess clinical response markers such as heart failure hospitalizations and mortality as well.

## Conclusion

LBBAP improved DVR significantly in HF patients. Post-implant TpTe was associated with the echocardiographic response after LBBAP among patients with wide QRS, especially for non-LBBB patients.

## Data Availability

The raw data supporting the conclusions of this article will be made available by the authors, without undue reservation.
